# Bacterial septicemia and herpesvirus infection in Antarctic fur seals (*Arctocephalus gazella*) stranded in the São Paulo coast, Brazil

**DOI:** 10.1007/s11259-024-10408-x

**Published:** 2024-06-01

**Authors:** Aricia Duarte-Benvenuto, A. M. Sánchez-Sarmiento, A. C. Ewbank, R. Zamana-Ramblas, S. Costa-Silva, N. Silvestre, T. Faita, L. B. Keid, R. M. Soares, C. F. Pessi, J. R. Sabbadini, M. F. Borges, R. B. Ferioli, M. Marcon, C. B. Barbosa, N. C. C. A. Fernandes, P. Ibáñez-Porras, P. E. Navas-Suárez, J. L. Catão-Dias, Carlos Sacristán

**Affiliations:** 1 Faculdade de Medicina Veterinária e Zootecnia – Universidade de São Paulo, São Paulo, SP Brazil; 2 Instituto Argonauta para a Conservação Costeira e Marinha, Ubatuba, SP Brazil; 3 Centro de Investigación en Sanidad Animal (CISA-INIA), CSIC, Valdeolmos, Madrid, Valdeolmos Spain; 4 Universidade de São Paulo, Pirassununga, SP Brazil; 5 Instituto de Pesquisas Cananéia, Cananéia, SP Brazil; 6 Centro de Patologia, Instituto Adolfo Lutz, São Paulo, SP Brazil; 7 Curso de Medicina Veterinária, Centro Universitário – FAM, São Paulo, SP Brazil

**Keywords:** Atlantic Ocean, *Klebsiella oxytoca*, *Pseudomonas aeruginosa*, Pneumonia, Gammaherpesvirus, Pinnipeds

## Abstract

In August 2021, two juvenile male Antarctic fur seals (*Arctocephalus gazella*) stranded in the southeastern Brazilian coast and were referred to rehabilitation centers. The animals presented increased body temperature, prostration, respiratory distress and despite treatment died. A necropsy following a standardized protocol was performed, and formalin-fixed tissues were processed for microscopic examination. Samples were screened for morbillivirus, herpesvirus, and *Brucella* spp. by molecular analyses (PCR, RT-PCR). Bacteriological culture was performed in samples collected from the lungs, trachea, and lymph nodes of both cases. The main histopathologic findings were of infectious nature, including multifocal necrotizing and fibrinous mixed interstitial pneumonia, bronchiolitis, and bronchitis, with intralesional myriad bacteria associated with vascular fibrinoid necrosis. *Pseudomonas aeruginosa* was isolated from tracheal and lung swabs of Case 1, and *Klebsiella oxytoca* was found in nostril swabs, tracheobronchial lymph nodes, and lung of Case 2. Gammaherpesvirus infection was detected in both cases, and the sequences retrieved were classified into the genus *Percavirus*. All tested samples were PCR-negative for *Brucella* spp. and morbillivirus. We hypothesize that the deficient immunological status in association with starvation predisposed the reactivation of herpesvirus and secondary bacterial co-infections. To the authors’ knowledge, this is the first molecular detection of herpesvirus in an Antarctic pinniped. These findings reinforce that Otariid gammaherpesvirus circulating in the Southern Hemisphere are likely endemic in the *Arctocephalus* genus. This report contributes to the current knowledge of health aspects affecting wild pinnipeds, especially in the poorly studied Antarctic species.

**Supplementary Information:**

The online version contains supplementary material available at 10.1007/s11259-024-10408-x.

## Introduction

The Brazilian coast is influenced by different sea currents that promote high marine mammal diversity (Short and Klein 2016). Eight pinniped species (seals, fur seals and sea lions) have been found in Brazilian waters, despite the absence of breeding colonies (Rocha-Campos and Gusmão-Câmara 2011). The most frequent vagrant species are the South American fur seal (*Arctocephalus australis*), the South American sea lion (*Otaria flavescens*), and the subantarctic fur seal (*Arctocephalus tropicalis*), which mainly occur in southern regions during austral winter, with few records of Antarctic fur seal (*Arctocephalus gazella*) (Rocha-Campos and Gusmão-Câmara 2011). *Arctocephalus gazella* has a wide geographic range and is the only fur seal species that breeds in colonies located southern of the Antarctic Convergence (Bonner 1981). The Antarctic region is zoological and geographically isolated; thus, the introduction of pathogens and parasites may have detrimental effects on local wildlife (Smeele et al. 2018). Therefore, monitoring infectious diseases of Southern Ocean fauna is essential for its conservation.

*Orthoherpesviridae* comprises enveloped double-stranded DNA viruses which can establish latency and are subdivided into three subfamilies: *Alphaherpesvirinae*, *Betaherpesvirinae*, and *Gammaherpesvirinae* (Gatherer et al. 2021). Alpha- and gammaherpesviruses have been detected in pinnipeds of the Southern Hemisphere, including *A. australis, A. tropicalis* and *O. flavescens* (Sacristán et al. 2018, 2021; Reisfeld et al. 2019a; Duarte-Benvenuto et al. 2022). In Antarctic pinnipeds, despite several serological evidence of neutralizing antibodies against alphaherpesvirus (Smeele et al. 2018), to this date, herpesvirus infection had not been molecularly confirmed.

The aim of this study is to report the clinic, pathologic and molecular findings of two *A. gazella* stranded in the São Paulo coast, southeastern Brazil, in 2021.

## Materials and methods

In August 2021, two *A. gazella* stranded in Ilha Comprida (Case 1) and São Sebastião (Case 2), in São Paulo state (Brazil) (Supplementary Fig. 1) and were transported to aquatic mammal rehabilitation facilities where underwent physical examination, hematologic, and radiographic analyses. Despite treatment, Case 1 died after fourteen days under rehabilitation, and Case 2 died within 24 h. Standardized post-mortem examinations were conducted and representative tissue samples of each case were collected in duplicate fixed in 10% neutral buffered formalin for histopathology or frozen at -20/-80ºC for PCR. Formalin-fixed-paraffin-embedded tissues were cut at 5 μm-thick and stained with hematoxylin-eosin, Gram and Ziehl-Neelsen for microscopic examination. Sterile swabs were collected from lungs, tracheobronchial and pulmonary lymph nodes, trachea from Case 1 and Case 2, and nostril from Case 2, and stored in AMIES/Stuart transport culture medium for microbiology.

Total DNA was extracted from ocular swab, blood, spleen, tongue, heart, liver, kidney, brainstem, lungs, trachea, and pulmonary and mesenteric lymph nodes from Case 1, and blood, heart, tongue, kidney, brainstem, lungs, and mesenteric lymph node from Case 2 (Supplementary Table), using the DNeasy Blood & Tissue kit (Qiagen, Valencia, USA), according to manufacturer’s instructions. RNA extraction of brainstem, spleen, liver, spinal cord, lung, mesenteric and pulmonary lymph nodes of both cases was performed with TRIzol-LS (Life Technologies Corporation, Rockville, USA). Extracted DNA was tested by broad-spectrum nested PCRs to partially amplify the herpesviral DNA polymerase (DPOL) and glycoprotein B (gB) genes (Sacristán et al. 2018). DNA samples were also screened for *Brucella* spp. by endpoint-PCR targeting the IS711 gene (Batinga et al. 2018). RNA samples were screened for morbillivirus by RT-PCR to amplify the phosphoprotein gene (Barrett et al., 1993). Previously confirmed samples for morbillivirus, *Brucella* spp., and herpesvirus were used as positive controls and DPEC water was used as negative control. Positive amplicons were confirmed by Sanger sequencing. The retrieved sequences were assembled in Mega7.0 using ClustalW alignment and compared with those available in GenBank by BLASTn search. Nucleotide and amino acid similarities to the closest sequences were calculated based on p-distance, after excluding primers. Maximum likelihood phylograms were constructed in Mega 7.0 with 1000 bootstrap replicates. Bootstrap values less than 70 were omitted.

**Table 1:** Main pathologic findings of the Antarctic fur seals (*Arctocephalus gazella*) stranded in the southeastern coast of São Paulo in 2021

Case No.	Gross findings	Microscopic findings
1	**External examination**: focal cutaneous ulceration on the right oral commissure. **Oral cavity**: multifocal ulcerative lesions on mandibular and maxillary gingiva. Focal ulcerative lesion (1 cm in diameter) on the dorsal aspect of the tongue. Dental tartar. **Nasal sinus**: mild naso-oropharyngeal acariasis by *Orthohalarachne attenuate* associated with hyperemia. **Subcutaneous and muscular tissue**: marked appendicular and axial muscle atrophy. Focal hemorrhage (6 cm in diameter) and edema on scapular region. **Lungs**: marked congestion, mild to moderate pulmonary edema, moderate suppurative bronchopneumonia on cranial lobes. **Trachea**: marked multifocal to coalescent ulcerative lesions. **Axillary and mesenteric lymph nodes**: moderate lymphadenomegaly. **Central nervous system**: mild to moderate cerebral edema. **Stomach and intestines**: gastrointestinal hyperemia and moderate presence of coagulated blood.	**Spleen**: moderate white pulp depletion. Moderate multifocal hemosiderosis. Mild to moderate multifocal reactive mesothelial cells. **Tongue**: marked focally extensive necrotizing glossitis with intralesional myriad bacteria (gram-negative coccobacilli), associated with vascular fibrinoid necrosis, and thrombosis. **Heart**: moderate multifocal cardiomyocyte degeneration and necrosis. **Small intestine**: mild to moderate multifocal mononuclear enteritis. **Lymph node**: moderate multifocal necrotizing lymphadenitis with intralesional bacteria (gram-negative coccobacilli) associated with vascular fibrinoid necrosis, and thrombosis. Mild multifocal hemosiderosis. **Lungs**: moderate multifocal necrotizing and fibrinous mixed interstitial pneumonia, bronchiolitis, and bronchitis, with intralesional myriad bacteria (gram-negative coccobacilli, and gram-positive cocci) associated with vascular fibrinoid necrosis, and thrombosis. Mild to moderate multifocal acute alveolar hemorrhage. Moderate alveolar edema and congestion. **Trachea**: moderate to marked multifocal necrotizing and fibrinous mixed tracheitis with intralesional bacteria (gram-negative coccobacilli) associated with vascular fibrinoid necrosis, thrombosis, and cartilage necrosis. **Kidney, thyroid, esophagus, liver, pancreas, lymph node, testicle, urinary bladder, spine, brain**: NSFO.
2	**External examination**: presence of diarrheic fetid feces on perianal region and mild mucopurulent discharge on the nostrils. **Subcutaneous tissue (inguinal region)**: mild multifocal presence of unidentified parasitic cysts. **Lungs**: marked congestion, mild to moderate pulmonary edema, and scattered presence of thin, firm whitish nodules. **Submandibular, axillary, mesenteric and pancreatic lymph nodes**: multicentric lymphadenomegaly, firm to granular white-yellow to dark nodules on parenchyma upon incision. **Retropharyngeal lymph nodes**: hemorrhagic appearance. **Central nervous system**: mild to moderate cerebral congestion with scattered petechiae (more evident at the brainstem). **Stomach and small intestine**: mild infestation of *Contracaecum ogmorhini*. **Large intestine**: mild infestation of *Corynosoma austral.*	**Lung**: moderate to marked multifocal necrotizing, fibrinous and hemorrhagic mixed interstitial pneumonia, bronchiolitis, and bronchitis, with intralesional myriad bacteria (gram-negative coccobacilli) associated with vascular fibrinoid necrosis, and thrombosis. Moderate multifocal acute alveolar hemorrhage. Mild to moderate alveolar edema and congestion. Medial mild multifocal arterial hypertrophy/hyperplasia. **Spleen**: moderate white pulp depletion. Mild to moderate multifocal reactive mesothelial cells. **Heart**: mild multifocal acute myocardial hemorrhage. **Peripancreatic, pre-scapular, mesenteric, axillar lymph nodes**: moderate multifocal necrotizing lymphadenitis with vascular fibrinoid necrosis, and thrombosis. Mild to moderate multifocal hemosiderosis. **Central nervous system**: *Pons*: mild multifocal perivascular acute hemorrhage. *Thalamus*: Moderate multifocal perivascular acute hemorrhage. Mild multifocal neutrophilic perivascular cuffing. *Occipital cortex and Cerebellum*: NSFO. **Pituitary gland**: anterior lobe (adenohypophysis) moderate to marked congestion. **Kidney**: mild to moderate acute tubular necrosis. Mild multifocal cortical acute hemorrhage. Mild focal mononuclear pyelitis. Mild multifocal tubular medullar mineralization. **Liver**: moderate to marked hepatocellular atrophy. Moderate pan lobular acute hemorrhage and necrosis. **Small intestine**: mild multifocal neutrophilic enteritis. **Skin**: mild to moderate focally extensive mononuclear dermatitis with moderate fibroplasia. **Adrenal glands, submandibular salivary gland, spine, esophagus, lumbar lymph node, skeletal muscle, thyroid, parathyroid, trachea, tongue**: NSFO.

*NSFO: No significant findings observed

## Results

Both Antarctic fur seals were juvenile males in poor nutritional condition. Upon admission, Case 1 (August 14th, 2021) was 20.1 kg and responsive to physical restraint. The respiratory frequency was 8 breaths/min with normal pulmonary auscultation, the heart rate of 148 bpm and body temperature of 34.4 °C. The fur seal presented pale oral mucosa and an abrasive lesion on right oral commissure. Two days prior to death (August 22th, 2021), it was prostrated, with 37.7 °C body temperature, 12 breaths/min and respiratory rattling, affecting mainly the right lung. The individual presented a 1.2 kg weight loss despite adequate nutrition and supplementation. For Case 2 (August 25th, 2021) upon initial assessment, the fur seal weighed 54 kg, was prostrated and poorly responsive to handling. The admission exam revealed a respiratory frequency of 5 breaths/min, sinus arrhythmia, and moderate dry rattles (crackling) on the left caudal lung. The heart rate was 58 bpm and the body temperature was 36.2 °C. The individual presented moderate dehydration, congested oral mucosa, and a capillary refill time of 4 s. Fetid diarrhea and an abrasive lesion exposing bone and muscle were noted at the distal limit of the tail. Despite treatment, less than 24 h after admission the animal developed fever of 39.2 °C, dyspnea, and died.

Radiographic examination of Case 2 revealed increased pulmonary radiopacity with a bronchial pattern on the left flank, suggestive of inflammatory/infectious process (Supplementary Fig. 1). Case 1 presented no radiographic changes (August 21th 2021). Upon admission, the hematological parameters of Case 1 indicated normocytic normochromic anemia (RBC = 3.3 × 10^6^/mm^3^, MCV = 96.97 fL, Hb = 11.74 g/dL) and leukopenia (7700/µl) with lymphopenia (154/µl). Case 2 featured marked macrocytic anemia (RBC = 1.60 × 10^6^/mm^3^, MCV = 312.50 fL, Hb = 8.8 g/dL), and leukocytosis (21,945/µl) with neutrophilia (14,923/µl) and lymphocytosis (5,486/µl), and hypoglycemia (75 mg/dL) (Ruoppolo and Loureiro 2014).

**Fig. 1:**
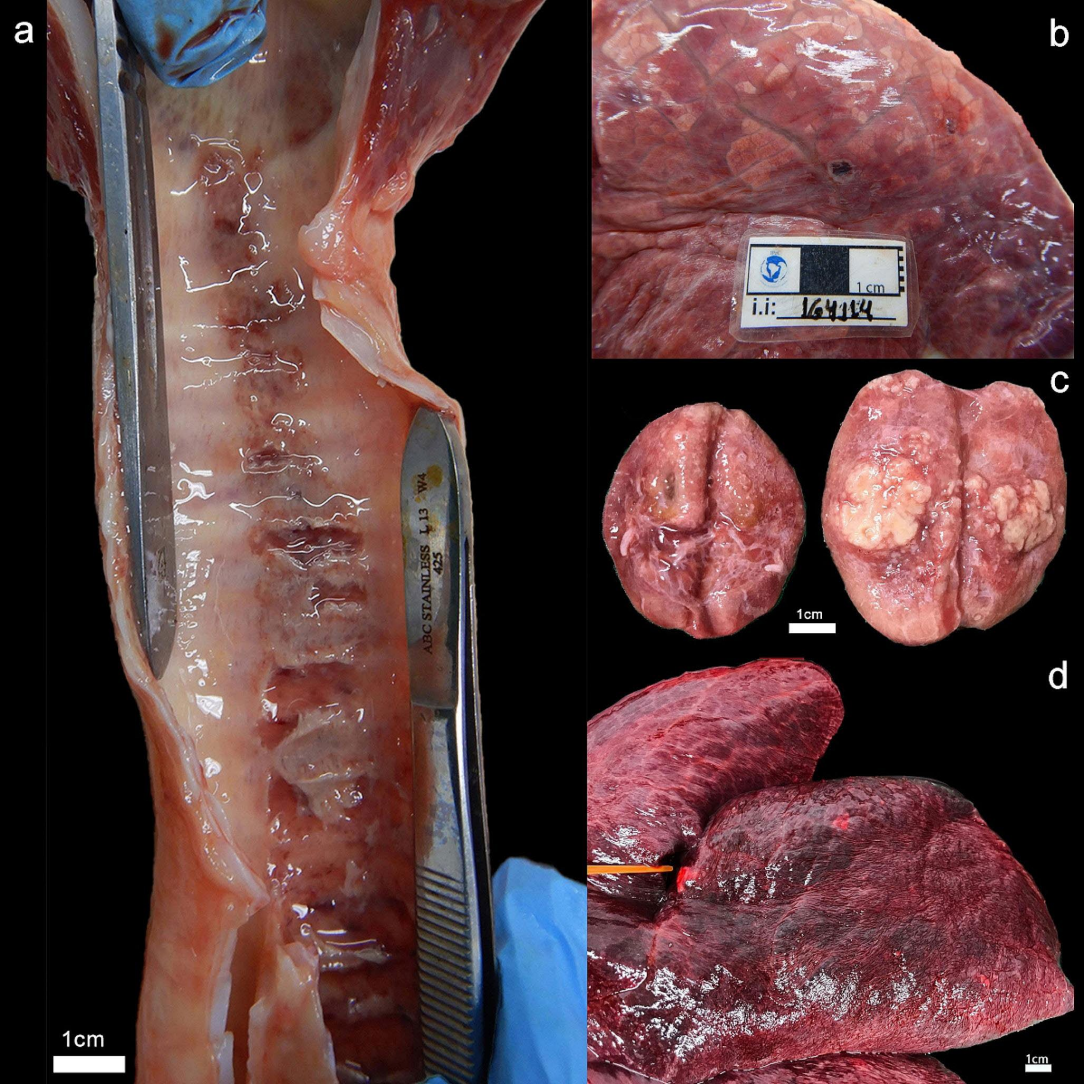
Gross findings of the Antarctic fur seals (*Arctocephalus gazella*): **Case 1.a.** Trachea. The mucosa exhibits multifocal to coalescent rounded ulcerated areas of dark reddish coloration (Severe multifocal fibrinonecrotic tracheitis). **b.** Lung. Note heterogeneous reddish coloration (lighter areas interspersed with darker areas) on the pleural surface, evidence of interlobular septa and a blackish focus with whitish edges and a depressed center (parenchymal necrosis). **Case 2.c**. Axillary lymph node. Note multiple lesions with defined borders protruding from the surface of the organ (at cut) with a whitish-yellowish coloration (Granulomatous lymphadenitis; also present in mesenteric, pre-scapular and pancreatic lymph nodes). **d.** Lung. Note a darker reddish coloration on the pleural surface, with two well-defined foci of whitish coloration (pulmonary congestion, pulmonary nodules)

**Fig. 2:**
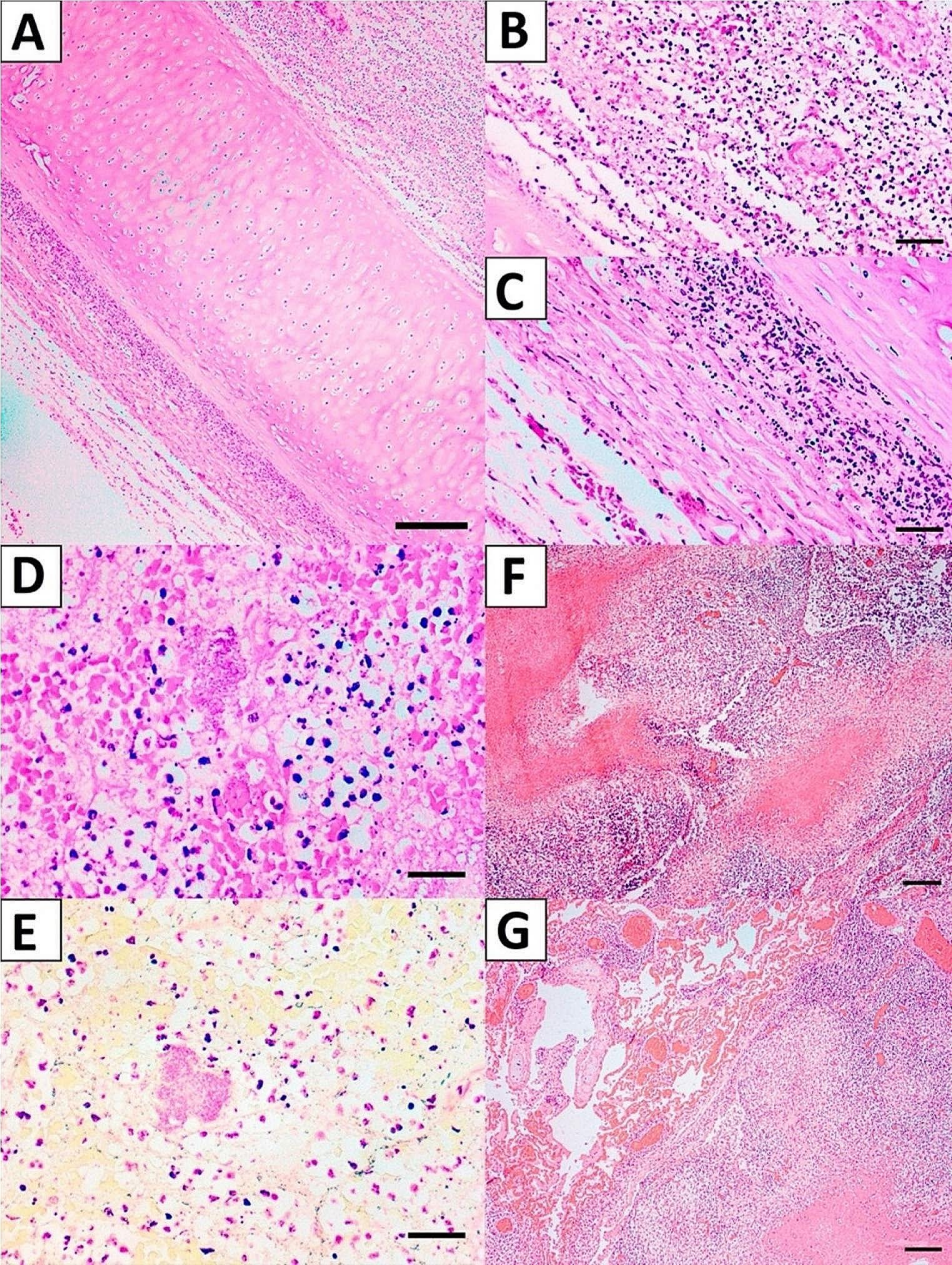
Microscopic findings of the Antarctic fur seals (*Arctocephalus gazella*): **Case (1) a**. Note exuberant infiltrate of inflammatory cells in the mucosa and serosa, HE, 4X. **b**. Note neutrophilic inflammatory infiltrate and fibrinoid necrosis of a small-caliber artery, HE, 20X. **c**. Note inflammatory infiltrate in the muscle layers and approaching the cartilage, HE, 20X. **d.** Note in the alveolar space a large number of erythrocytes, degenerated neutrophils, and fibrin deposits associated with a myriad of bacteria, HE, 40X. **e.** Note the Gram-negative characteristics of the bacterial myriad, HE, 40X. Case (2) **f.** Note exuberant multiple necrotic areas and infiltrate of inflammatory cells in the lymph node associated with vascular fibrinoid necrosis, and thrombosis. HE, 4X. **g.** Note exuberant infiltrate of inflammatory cells in the pulmonary parenchyma thickening the alveolar septum, associated with large necrotic areas, marked fibrin deposition, hemorrhage, alveolar edema and congestion, HE, 4X

All gross and histopathological findings are described in Table 1. The most significant gross lesions observed in both cases were in respiratory system (Fig. 1), comprising lungs and trachea. Microscopically, there was moderate multifocal necrotizing and fibrinous mixed interstitial pneumonia, bronchiolitis and bronchitis, with a myriad of intralesional bacteria associated with vascular fibrinoid necrosis, and moderate lymphoid depletion of white pulp (Fig. 2). In both cases, the most likely cause of death was bacterial septicemia. Special stains of tissue sections from both animals did not detect any Ziehl-Neelsen bacilli.

Two isolates of *Pseudomonas aeruginosa* resistant to penicillin class (i.e., ampicillin, amoxicillin/clavulanic acid), and 3rd generation cephalosporins (i.e., cefovecine, cephalexin) were identified in Case 1’ tracheal and lung swabs; intermediate resistance against tetracycline class was observed. Two isolates of *Klebsiella oxytoca* resistant to penicillin class (i.e., ampicillin, penicillin), aminoglycoside class (i.e., amikacin), and cephalosporin class (i.e., ceftiofur) were found in lung, tracheobronchial lymph nodes, and nasopharynx swabs of Case 2; intermediate resistance against fluorquinolone class (i.e.,enrofloxacin) was found.

All tested tissue samples were PCR-negative for *Brucella* spp. and morbillivirus. Herpesvirus DPOL gene was amplified in trachea, lung, and mesenteric lymph node collected from Case 1, and in lungs, mesenteric lymph node, and spleen of Case 2. Herpesvirus gB gene was also amplified in ocular swab, brainstem, lung, trachea, mesenteric and pulmonary lymph nodes, and tongue from Case 1, and in brainstem, blood, lung, spleen, and mesenteric lymph node sampled from Case 2. All molecular data are summarized in Supplementary Table 1.

All obtained sequences clustered in the DPOL and gB phylograms with other pinniped sequences within genus *Percavirus* (Supplementary Fig. 2A, B). Additionally, in the gB phylogram the consensus sequence obtained from brainstem, trachea, lung, and tongue of Case 1 and the sequence of Case 2 clustered with different otariid gammaherpesvirus 5 strains, while the sequences from kidney, mesenteric lymph node, lung, and blood of Case 1 grouped with otariid gammaherpesvirus 5. Representative gammaherpesvirus sequences were submitted to GenBank (OQ876251, OQ876252, OQ876253, OR086881 and OR086882).

## Discussion

The stranding of pinnipeds far from their breeding colonies is common and usually associated with the species’ natural dispersion patterns, likely influenced by prey availability, marine currents, and oceanographic events (Ruoppolo and Loureiro 2014). In Brazil, they usually occur during austral winter, and involve juvenile males - especially *Arctocephalus* specimens – which typically strand in poor body condition (Ruoppolo and Loureiro 2014; Sacristán et al. 2018; Reisfeld et al. 2019a, b; Duarte-Benvenuto et al. 2022), as observed herein.

Both individuals presented with anemia, a common finding in malnourished fur seals (Ruoppolo and Loureiro 2014; Sacristán et al. 2018; Reisfeld et al. 2019a, b; Duarte-Benvenuto et al. 2022), likely associated with starvation. Additionally, Case 1 presented with marked lymphopenia, suggesting immunodeficiency (Ruoppolo and Loureiro 2014), while Case 2 presented a leukogram consistent with infectious/inflammatory process, presumably due to pneumonia and possible secondary sepsis.

Both individuals presented with fever, respiratory distress, and extreme prostration prior to death. Based on the antemortem clinical pathology results and postmortem findings, these signs are likely related to agonal septicemia and systemic infectious disease. This suggestion is reinforced by the detection of gammaherpesvirus infection in multiple organs, presence of intralesional bacteria in the lungs and lymph nodes (Case 1 and 2), and trachea and tongue (Case 1), hematologic and radiographic alterations (Case 2), as well as histopathologic findings (Cases 1 and 2). We hypothesize that immunosuppression and generalized debilitation associated with starvation in both animals likely predisposed, and possibly exacerbated the herpesvirus infection with subsequent secondary bacterial involvement, resulting in death.

The fur seals had bacterial interstitial pneumonia, accompanied by the identification of multidrug-resistant *P. aeruginosa* and *K. oxytoca* in Case 1 and 2, respectively. Both gram-negative bacteria are widespread within the environment and may comprise part of the normal gastrointestinal flora in some marine mammal species. The bacteria are considered opportunistic pathogens in mammals, and a major cause of illness in humans with immunosuppressive and chronic conditions (Podschun and Ullmann 1998; Diggle and Whiteley 2020). In pinnipeds, *P. aeruginosa* has been reported in grey seals (*Halichoerus grypus*) and *Z. californianus* with corneal ulcers (Fleming and Bexton 2016; Simeone et al. 2017), and has been recovered from cutaneous abscesses in Galapagos sea lions (*Zalophus wollebaeki*) (Rand 1975). Nevertheless, it has also been detected in oral swabs of clinically healthy *Z. californianus*, suggesting it could be part of the normal microbiota of the upper digestive and respiratory tracts of otariids (Zavala-Norzagaray et al. 2022). The descriptions of *K. oxytoca* in pinnipeds are scarce, limited to its isolation in seven New Zealand sea lions (*P. hookeri*) and in a hooded seal (*Cystophora cristata*) without reported associated lesions (Bogomolni et al. 2008; Michael et al. 2021). Herein, the escalating bacterial infection and development of septicemia in both cases was linked to the poor immune status. Future studies investigating the presence and potential pathogenicity of *P. aeruginosa* and *K. oxytoca* in fur seals are warranted.

The detected gammaherpesviruses gB were highly similar to those previously described in stranded *A. tropicalis* in Brazil (Reisfeld et al. 2019a; Duarte-Benvenuto et al. 2022), suggesting virus exchange among pinniped species, likely in the breeding colonies shared by these species (Hofmeyr et al. 2006). Otariid gammaherpesvirus 5, 6, and 7 have been reported in stranded fur seals in Brazil, and are likely endemic in the genus *Arctocephalus* (Sacristán et al. 2018, 2021; Reisfeld et al. 2019a; Duarte-Benvenuto et al. 2022). The gammaherpesvirus DPOL sequences were closer to otariid gammaherpesvirus 2 obtained in *Z. californianus* of California, USA, possibly due to the limited number of similar sequences from pinnipeds of the Southern Hemisphere. Case 1 was under human care for 14 days, and during that period, shared the facility with two *A. australis*. Nevertheless, herpesvirus transmission among these specimens was ruled out, once Case 1’s ocular swab tested positive for gammaherpesvirus upon admission, indicating a natural infection prior to its entry. Those findings highlight the importance of screening for infectious agents upon entrance in the facilities, and implementing biosecurity and quarantine procedures in order to avoid cross-infection, even of potential endemic agents as described herein.

Despite their usually endemic and subclinic characteristics, herpesvirus infections have been associated with lesions and even death in immunosuppressed and young individuals (Duarte-Benvenuto et al. 2022). Thus, considering the escalating impact of climate change within the Antarctic ecosystem, its consequent repercussions over prey distribution and top predators density (e.g., pinnipeds), and its potential effect on these individuals’ immune status, we highlight the importance of surveying herpesviruses in other pinniped species present in that continent (e.g., southern elephant seal [*Mirounga leonina*], crabeater seal [*Lobodon carcinophaga*]), and further investigation of its prevalence in *A. gazella.*

## Conclusion

Herein we described two cases of systemic gammaherpesvirus infection and bacterial septicemia in *A. gazella* stranded in the Brazilian coast. To the authors’ knowledge, this is the first molecular detection of herpesvirus in an Antarctic pinniped. Our findings reinforce that otariid gammaherpesviruses (especially 5, 6, and 7) are circulating in the Southern Hemisphere and are likely endemic to *Arctocephalus* genus. This report broadens the current knowledge on clinicopathological features associated with herpesvirus infection, multidrug resistant bacterial co-infections, and health conditions affecting Antarctic pinniped species. The information derived from this study will better inform management and care of pinnipeds under human care.

### Supplementary information

Below is the link to the electronic supplementary material.

Supplementary Table 1 (DOCX 15.9 KB)

Supplementary Fig. 1 Map showing the stranding location of the Antarctic fur seals (*Arctocephalus gazella*) included in the study. Source of the map: own source. (PNG 374 KB)

High Resolution Image (TIF 528 KB)

Supplementary Fig. 2 Radiographic analyses of Case 2. a, b. Dorsoventral projections of thorax and pelvis. Note increased pulmonary radiopacity with a bronchial pattern on the left flank, more evident in the topography of the caudal lung lobes (a). (JPEG 875 KB)

Supplementary Fig. 3 DNA polymerase (A) and glycoprotein B (B) maximum likelihood phylograms of the alignment of the deduced amino acid gammaherpesvirus consensus sequences obtained in Antarctic fur seals (*Arctocephalus gazella*, green dots), in other pinnipeds and gammaherpesvirus species (of different genera) recognized by the International Committee of Taxonomy of Viruses. Phocid alphaherpesvirus 1 sequences were selected as outgroups for both phylograms. The model selected for both phylograms was Le Gascuel with a discrete gamma distribution. The reliability of the phylograms was tested by 1000 replicate bootstrap analyses omitting values below 70. (JPEG 2.72 MB)

## Data Availability

No datasets were generated or analysed during the current study.
