# Supplementary material for: Bacterial septicemia and herpesvirus infection in Antarctic fur seals (Arctocephalus gazella) stranded in the São Paulo coast, Brazil
Source: Vet Res Commun. 2024 Jun 1;48(4):2819–26. doi: 10.1007/s11259-024-10408-x (PMC11315713; doi:10.1007/s11259-024-10408-x)
Supplement: Supplementary file 1 — (DOCX 15.9 KB) [file 11259_2024_10408_MOESM1_ESM.docx]

**Table 2.** Molecular data of gammaherpesviruses detected in *A. gazella* stranded in São Paulo coast in August 2021.

| **Case** | **Tissue sample** | **DPOL identities** | | **gB identities** | |
| --- | --- | --- | --- | --- | --- |
|  |  | **nt** | **aa** | **nt** | **aa** |
| 1 | Brainstem | NA | NA | 99.34% with otariid gammaherpesvirus 5 (MZ147499) of a subantarctic fur seal ( *Arctocephalus tropicalis)* | 100% with otariid gammaherpesvirus 5 (MZ147499) of a subantarctic fur seal |
| 1 | Tongue | NA | NA |  |  |
| 1 | Lungs | 98.8% to otariid gammaherpesvirus 2 (GenBank accession no. GQ429148) of a California sea lion (*Zalophus californianus*) | 98.8% to otariid gammaherpesvirus 2 (GQ429148) of a California sea lion |  |  |
| 1 | Trachea |  |  |  |  |
| 1 | Mesenteric lymph node |  |  | 99.1% with otariid gammaherpesvirus 7 (MH921584) of a subantarctic fur seal | 99.3% with otariid gammaherpesvirus 7 (MH921584) of a subantarctic fur seal |
| 1 | Blood | NA | NA |  |  |
| 1 | Kidney | NA | NA |  |  |
| 1 | Pulmonary lymph node | NA | NA |  |  |
| 2 | Blood | NA | NA | 96.7% with otariid gammaherpesvirus 5 (MZ147499) of a subantarctic fur seal | 100% with otariid gammaherpesvirus 5 (MZ147499) of a subantarctic fur seal |
| 2 | Lungs | 98.8% to otariid gammaherpesvirus 6 (MW264415) of a South American fur seal (*Arctocephalus australis)* | 98.2% to otariid gammaherpesvirus 6 (MW264415) of a South American fur seal |  |  |
| 2 | Spleen |  |  |  |  |
| 2 | Mesenteric lymph node |  |  | NA | NA |
