# Supplementary figures and images for: Bacterial septicemia and herpesvirus infection in Antarctic fur seals (Arctocephalus gazella) stranded in the São Paulo coast, Brazil
Source: Vet Res Commun. 2024 Jun 1;48(4):2819–26. doi: 10.1007/s11259-024-10408-x (PMC11315713; doi:10.1007/s11259-024-10408-x)

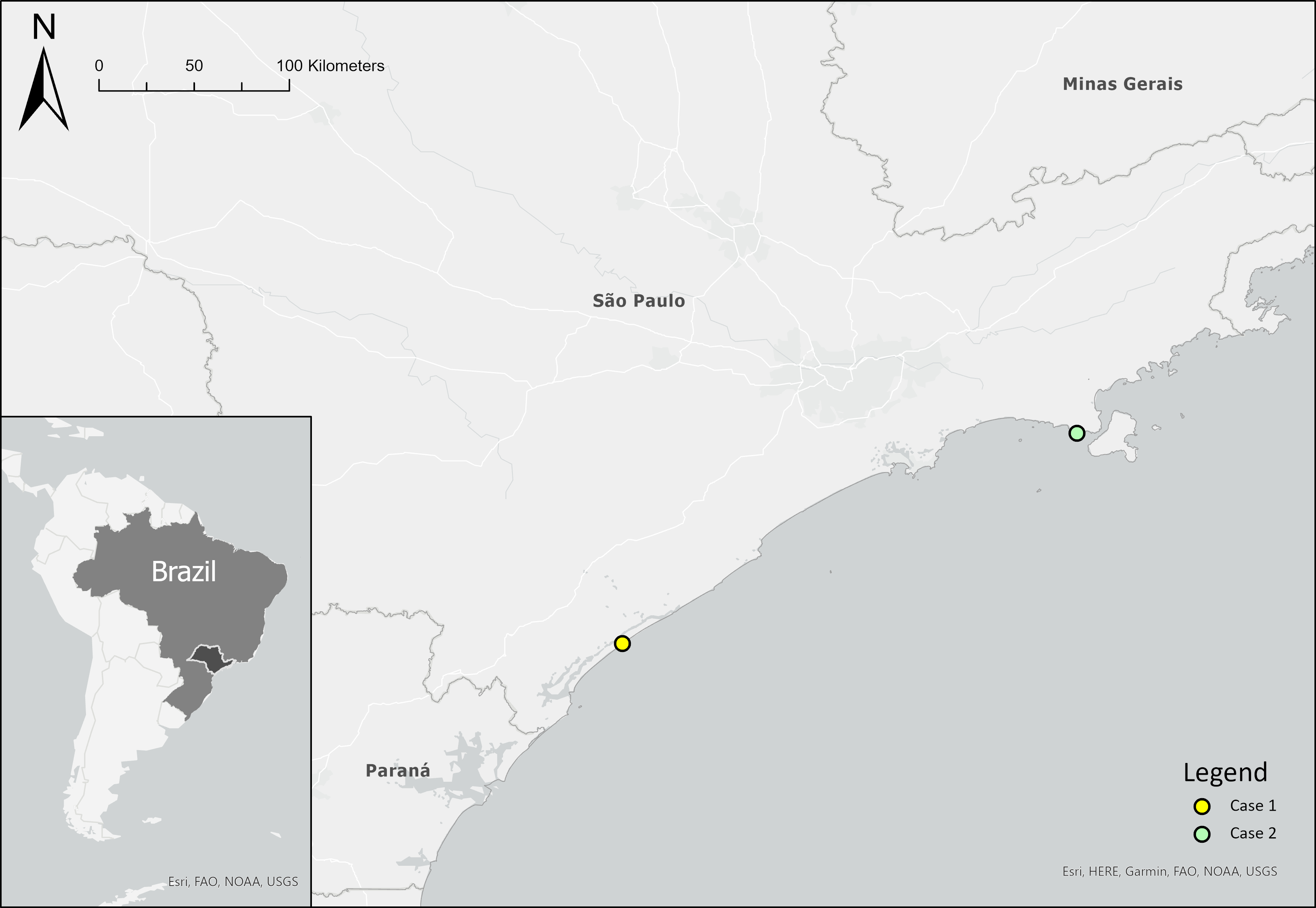

Supplement: Supplementary file 2 — Map showing the stranding location of the Antarctic fur seals (Arctocephalus gazella) included in the study. Source of the map: own source. (PNG 374 KB) [file 11259_2024_10408_Fig3_ESM.png]

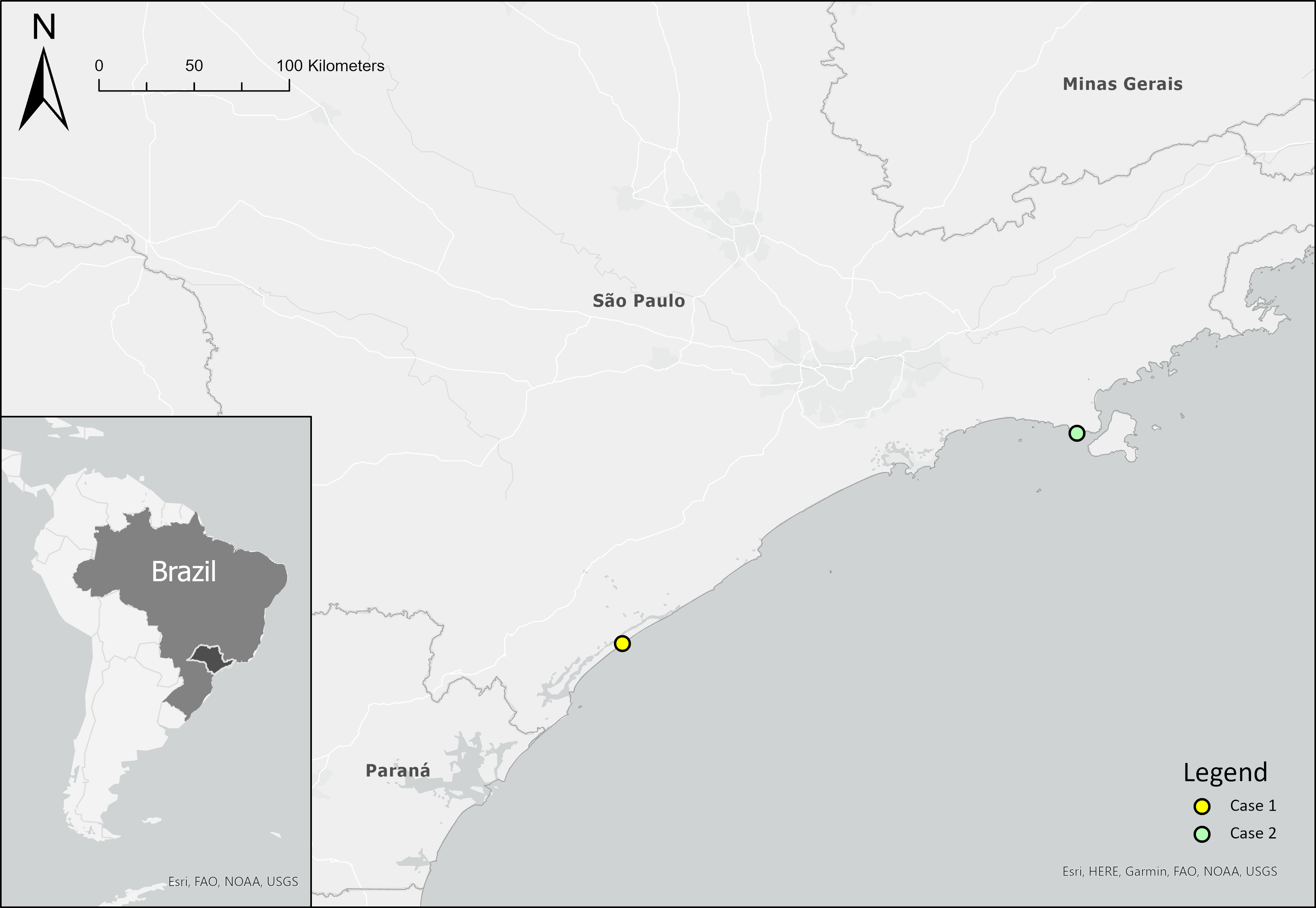

Supplement: Supplementary file 3 — High Resolution Image (TIF 528 KB) [file 11259_2024_10408_MOESM2_ESM.tif]

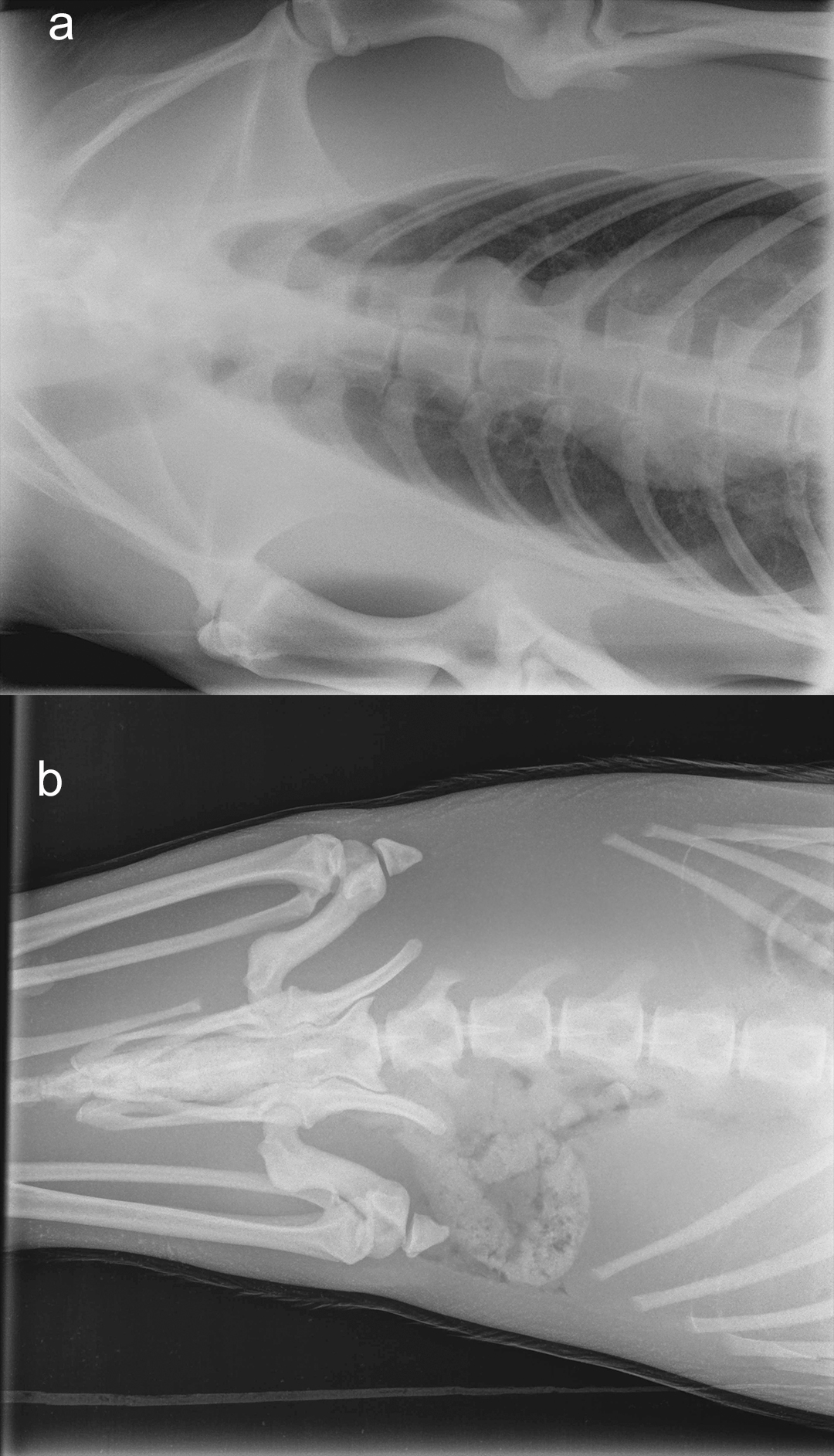

Supplement: Supplementary file 4 — Radiographic analyses of Case 2. a, b. Dorsoventral projections of thorax and pelvis. Note increased pulmonary radiopacity with a bronchial pattern on the left flank, more evident in the topography of the caudal lung lobes (a). (JPEG 875 KB) [file 11259_2024_10408_MOESM3_ESM.jpeg]

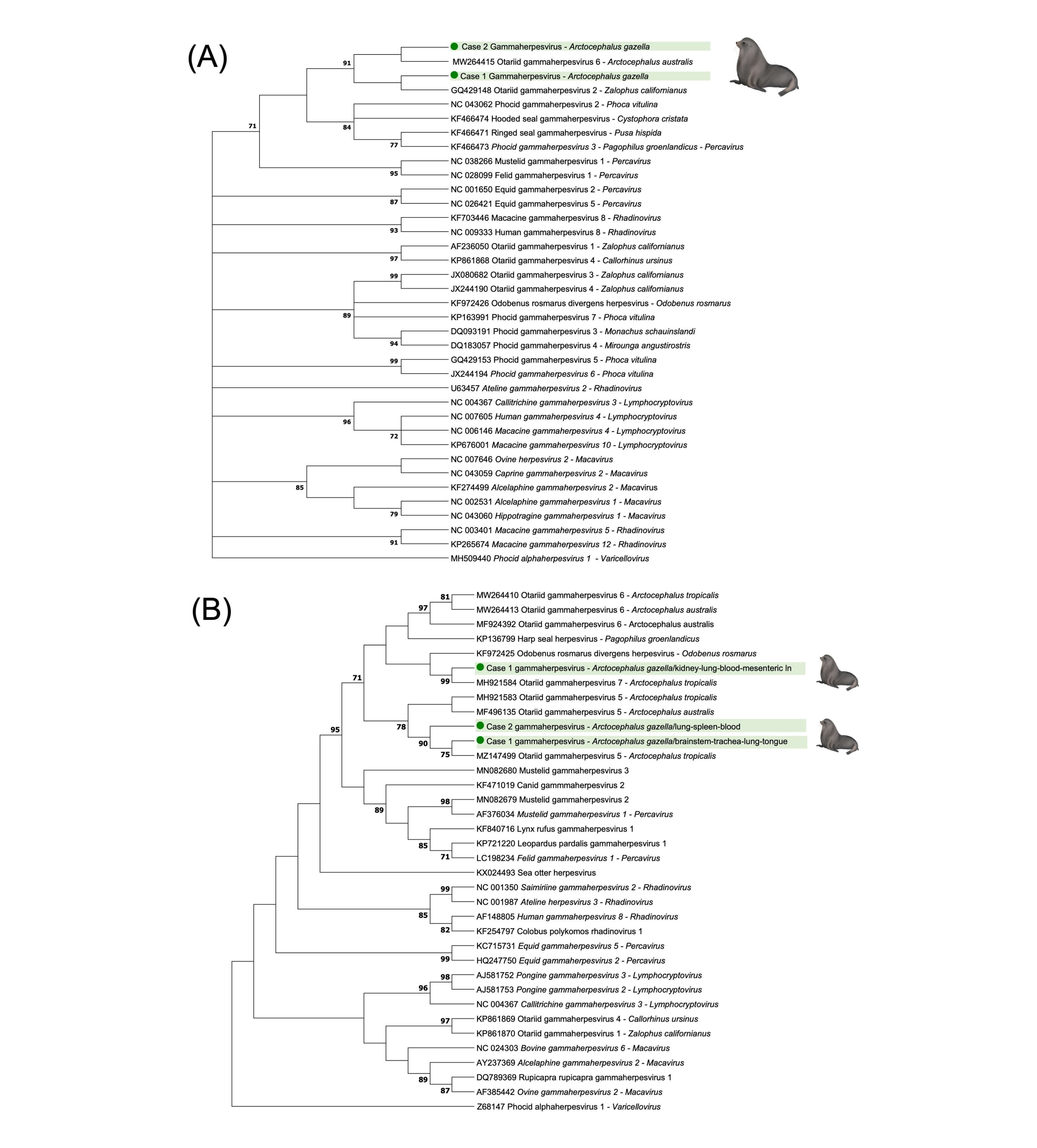

Supplement: Supplementary file 5 — DNA polymerase (A) and glycoprotein B (B) maximum likelihood phylograms of the alignment of the deduced amino acid gammaherpesvirus consensus sequences obtained in Antarctic fur seals (Arctocephalus gazella, green dots), in other pinnipeds and gammaherpesvirus species (of different genera) recognized by the International Committee of Taxonomy of Viruses. Phocid alphaherpesvirus 1 sequences were selected as outgroups for both phylograms. The model selected for both phylograms was Le Gascuel with a discrete gamma distribution. The reliability of the phylograms was tested by 1000 replicate bootstrap analyses omitting values below 70. (JPEG 2.72 MB) [file 11259_2024_10408_MOESM4_ESM.jpg]
